# Pharmacogenetic Factors Affecting Asthma Treatment Response. Potential Implications for Drug Therapy

**DOI:** 10.3389/fphar.2019.00520

**Published:** 2019-05-21

**Authors:** Jesús Miguel García-Menaya, Concepción Cordobés-Durán, Elena García-Martín, José A. G. Agúndez

**Affiliations:** ^1^Allergy Service, ARADyAL Instituto de Salud Carlos III, Badajoz University Hospital, Badajoz, Spain; ^2^Allergy Service, ARADyAL Instituto de Salud Carlos III, Hospital de Mérida, Mérida, Spain; ^3^ARADyAL Instituto de Salud Carlos III, University Institute of Molecular Pathology Biomarkers, Universidad de Extremadura, Cáceres, Spain

**Keywords:** asthma, precision medicine, corticosteroids (CORT), anti-leukotrienes, beta-agonists, biologic agents, pharmacogenenomics and personalized medicine

## Abstract

Asthma is a frequent disease, mainly characterized by airway inflammation, in which drug therapy is crucial in its management. The potential of pharmacogenomics testing in asthma therapy has been, to date, little explored. In this review, we discuss pharmacogenetic factors affecting asthma treatment, both related to drugs used as controller medications for regular maintenance, such as inhaled corticosteroids, anti-leukotriene agents, long-acting beta-agonists, and the new biologic agents used to treat severe persistent asthma. In addition, we discuss current pharmacogenomics knowledge for rescue medications provided to all patients for as-needed relief, such as short-acting beta-agonists. Evidence for genetic variations as a factor related to drugs response has been provided for the following genes and groups of drugs: Inhaled corticosteroids: *FCER2*; anti-leukotriene agents: *ABCC1*, and *LTC4S*; beta-agonists: *ADRB2*. However, the following genes require further studies confirming or rejecting association with the response to asthma therapy: *ADCY9, ALOX5, ARG1, ARG2, CRHR1, CRHR2, CYP3A4, CYP3A5, CYSLTR1, CYSLTR2, GLCCI1, IL4RA, LTA4H, ORMDL3, SLCO2B1, SPATS2L, STIP1, T, TBX21, THRA, THRB*, and *VEGFA*. Although only a minority of these genes are, at present, listed as associated with drugs used in asthma therapy, in the Clinical Pharmacogenomics Implementation Consortium gene-drug pair list, this review reveals that sufficient evidence to start testing the potential of clinical pharmacogenomics in asthma therapy already exists. This evidence supports the inclusion in pilot pharmacogenetics tests of at least four genes. Hopefully these tests, if proven useful, will increase the efficiency and the safety of asthma therapy.

## Introduction

Asthma is an important and frequent disease, mainly characterized by airway inflammation. Asthma definition includes a history of respiratory symptoms such as wheezing, shortness of breath, chest tightness, and cough that vary over time and in intensity, together with variable expiratory airflow limitation [Global Initiative for Asthma (GINA), 2018]. Asthma has worldwide relevance, and it has been estimated that the number of affected individuals is over 330 million (Vos et al., 2012). In the developed world, between 5 and 10% of the adult population suffer from asthma (Masoli et al., 2004). It is estimated that 346,000 deaths are caused by asthma worldwide every year (Lozano et al., 2012). The main aims of asthma treatment are good symptomatic control, and reduction in future risks, including fixed airflow limitation.

Drugs used in asthma treatment are classified into two main groups. Controller drugs: These drugs are used for symptom control, reducing inflammation in the airways, and preventing complications such as lung function deterioration, or symptom exacerbations. The second group is classified as relievers or rescue drugs: These drugs are prescribed for relief of symptoms as-needed, for example when exacerbations or aggravation of asthma exist [Global Initiative for Asthma (GINA), 2018]. In order to achieve these goals, international and national guides have been published [Plaza Moral and Comité Ejecutivo de GEMA, 2016; Global Initiative for Asthma (GINA), 2018].

Despite improvements in treatment and the implementation of these guides, adequate control of symptoms is not achieved for more than half of patients (Chapman et al., 2008). There are several reasons for this. About 10% of asthmatic patients are refractory to the treatment, and severe asthma causes a relevant socio-economic burden (Maio et al., 2018). Sometimes the reason is suboptimal treatment adherence (Dekhuijzen et al., 2018). In addition, there are comorbidities, such as cigarette smoking, that may influence asthma treatments by impairing the response to corticosteroids (both inhaled and oral) (Chaudhuri et al., 2003), and perpetuating symptoms despite treatment (Cerveri et al., 2012). It has been reported that the response of patients to standard asthma therapy, or asthma adequate control, can be affected by obesity (Peters-Golden et al., 2006; Rodrigo and Plaza, 2007). In addition, genetic variations not related to drug metabolism or action may modify the clinical manifestations of asthma, for instance, those related to histamine metabolism (García-Martín et al., 2007). An important group of causes explaining different responses to asthma medications is related to the expression of drug targets and/or enzymes involved in drug biodisposition, often due to genetic variability (Ortega et al., 2015).

**Figure 1:**
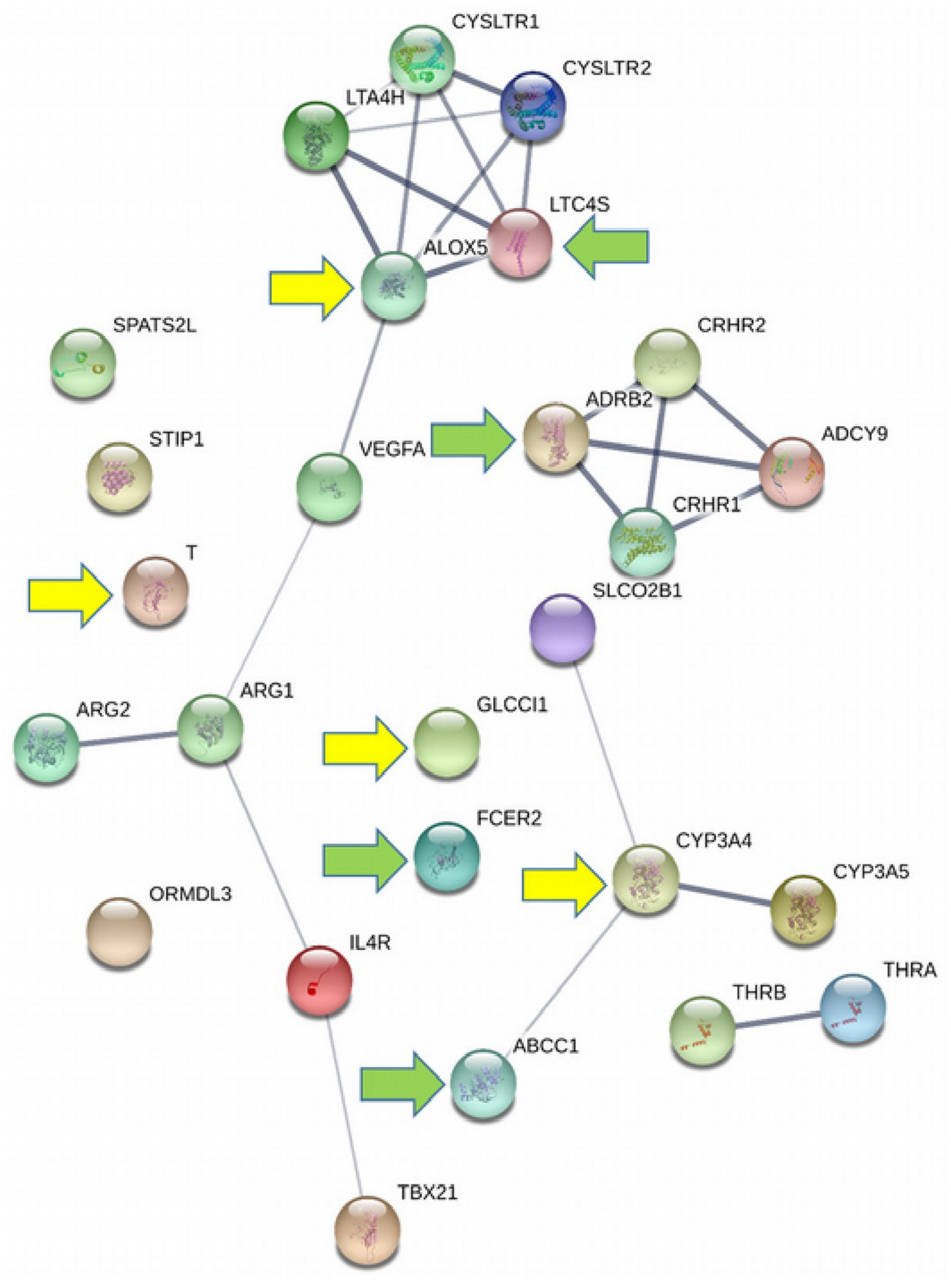
Interactions between genes putatively related to the response to asthma therapy. The line thickness indicates the strength of data support. Green arrows indicate the most promising genes for pharmacogenomics implementation and yellow arrows indicate promising genes that require further confirmation.

In this review, we analyze the above-mentioned group of causes, that is, the different polymorphisms affecting drug target molecules, or affecting drug metabolism and thus causing inter-individual differences in the response to asthma treatment and evolution. We review polymorphisms affecting response to different groups of controller and reliever medications. Because asthma seems to be a consequence of the interplay of genetics and environmental factors, the genetic variations discussed here hold great promise for the implementation of precision medicine in this disease. Figure 1 summarizes the crosstalk between the genes covered in this review, constructed with STRING (Szklarczyk et al., 2017), and the major associations identified with asthma therapy. Three major clusters appear, including one related to leukotrienes, another that includes *CRHR* genes (related with inhaled corticosteroids), and *ADRB2* and *ADCY9* (related with beta-agonists), and a third cluster related to drug-metabolizing enzymes and transporters. The remaining genes have weak or no crosstalk with the mentioned clusters. Details of the putative associations of these genes with response to asthma therapy are provided below.

## Drugs Used in Asthma Treatment

### Inhaled Corticosteroids

Inhaled corticosteroids (ICSs) constitute the main anti-inflammatory drug therapy in asthma. It has been demonstrated that ICSs have several benefits, such as improvement of symptoms, lung function, airway responsiveness, and quality of life. In addition, ICSs diminish airway inflammation and the risk of exacerbations and hospitalizations (Covar, 2016).

Corticotropin-releasing hormone receptor 1 is encoded by the *CRHR1* gene (Duong-Thi-Ly et al., 2017). Activation of the receptor by the corticotropin-releasing hormone (CRH) causes anti-inflammatory effects by stimulating cortisol production (Dautzenberg and Hauger, 2002). In 2004, Tantisira et al. demonstrated that variability in the *CRHR1* gene was associated with an increased response to ICSs therapy. The primary outcome measure of the association analyses was percent change in forced expiratory volume in 1 s (FEV1) over time in response to ICSs. By means of candidate gene studies, the authors observed that the single nucleotide variations (SNVs) rs242941 and rs1876828 were associated with positive treatment response and improved FEV1 in those populations (Tantisira et al., 2004b). However, these results were not replicated in three subsequent studies (Dijkstra et al., 2008; Rogers et al., 2009; Keskin et al., 2016) (see Table 1). Another study involving children (Mougey et al., 2013a) did replicate the findings by Tantisira et al. (2004b) with regard to the SNV rs1876828 but not for the SNV rs242941. Overall findings are, therefore, inconclusive so far, and further studies are required.

**Table 1:** Summary of the major findings related to pharmacogenetics factors affecting asthma treatment response.

**Gene**	**Polymorphism**	**Population, *n***	**Major clinical findings associated with the minor allele**.	**References**
**INHALED CORTICOSTEROIDS**				
*CRHR1*	rs242941 (C/A)	USA, Caucasians, *n* = 781	Positive response to ICSs treatment	Tantisira et al., 2004b
*CRHR1*	rs242941 (C/A)	Netherlands, *n =* 164	No association with improved FEV1 after ICSs treatment	Dijkstra et al., 2008
*CRHR1*	rs242941 (C/A)	USA, children, *n =* 311	Poor lung function response	Rogers et al., 2009
*CRHR1*	rs242941 (C/A)	Turkey, children, *n =* 82	No association with improved FEV1 after ICSs treatment	Keskin et al., 2016
*CRHR1*	rs242941 (C/A)	USA, adults and children, *n =* 129	Decrease of predicted FEV1	Mougey et al., 2013a
*CRHR1*	rs1876828 (C/T)	USA, Caucasians, *n =* 336	Higher FEV1 improvement	Tantisira et al., 2004b
*CRHR1*	rs1876828 (C/T)	Netherlands, *n =* 164	No FEV1 improvement after ICSs treatment	Dijkstra et al., 2008
*CRHR1*	rs1876828 (C/T)	Turkey, children, *n =* 82	No FEV1 improvement after ICSs treatment	Keskin et al., 2016
*CRHR1*	rs1876828 (C/T)	USA, adults and children, *n =* 129	Higher FEV1 improvement	Mougey et al., 2013a
*STIP1*	rs4980524 (A/C) rs6591838 (A/G) rs2236647 (C/T) rs2236648 (C/T) and rs1011219 (G/A)	USA, adults, *n =* 439	Lower FEV1 improvement	Hawkins et al., 2009
*TBX21*	rs2240017 (C/G)	USA, children, *n =* 1,041	Decreased airway responsiveness	Tantisira et al., 2004a
*TBX21*	rs2240017 (C/G)	Korea, adults, *n =* 53	Worse control during ICSs treatment	Ye et al., 2009
*TBX21*	rs9910408 (A/G)	Slovenia, adults, *n =* 208	Worse response to ICSs treatment	Lopert et al., 2013
*GLCCI1*	rs37972 (C/T)	USA, adults, *n =* 844 USA, children, *n =* 219	Reduced lung function in response to ICSs	Tantisira et al., 2011
*GLCCI1*	rs37973 (A/G)	Japanese, adults, *n =* 224	Reduced lung function in response to ICSs	Izuhara et al., 2014
*GLCCI1*	rs37972 (C/T) and rs37973 (A/G)	Chinese, adults, *n =* 182	Poorer improvement in FEV1 after ICSs treatment	Hu et al., 2016
*GLCCI1*	rs37973 (A/G)	Chinese, adults, *n =* 418	Poorer clinical response to ICSs	Xu et al., 2017
*GLCCI1*	rs37973 (A/G)	USA, adults and adolescents, *n =* 1,924	No FEV1 changes after ICSs treatment	Hosking et al., 2014
*GLCCI1*	rs37973 (A/G)	Slovenia, adults, *n =* 208	Better response to ICSs treatment	Rijavec et al., 2018
*T gene*	rs1134481 (G/T)	USA, Caucasians adults and children, *n =* 418	Worse FEV1 response to ICSs	Tantisira et al., 2012
*T gene*	rs2305089 (C/T)	USA, Caucasians adults and children, *n =* 418	Worse FEV1 response to ICSs	Tantisira et al., 2012
*T gene*	rs3099266 (C/T)	USA, Caucasians adults and children, *n =* 418	Worse FEV1 response to ICSs	Tantisira et al., 2012
*FCER2*	rs28364072 (A/G)	USA, children, *n =* 311	Severe exacerbation despite ICSs treatment	Tantisira et al., 2007
*FCER2*	rs28364072 (A/G)	USA, children, *n =* 311	Poorer lung function response after ICSs treatment	Rogers et al., 2009
*FCER2*	rs28364072 (A/G)	Netherlands, children, *n =* 1,325	More asthma-related hospitalizations after ICSs treatment	Koster et al., 2011
*ORMDL3*	rs2872507 (G/A)	Slovenia, children, *n =* 311	Better outcome in response to ICSs	Berce et al., 2013
*VEGFA*	rs2146323 (C/A)	Slovenia, children, *n =* 311	Better ICSs treatment response	Balantic et al., 2012
*CYP3A4*	rs35599367 (G/A)	USA, children, *n =* 734	Improved asthma control after ICSs treatment	Stockmann et al., 2013
**ANTI-LEUKOTRIENE AGENTS**				
*ALOX5*	Tandem repeats in the *ALOX5* core promoter	USA, adults,*n =* 221	Poorer FEV1 response	Drazen et al., 1999
*ALOX5*	Tandem repeats in the *ALOX5* core promoter	UK, adults, *n =* 52	No association with bronchodilator response	Fowler et al., 2002
*ALOX5*	Tandem repeats in the *ALOX5* core promoter	Spain, adults and adolescents, *n =* 61	More asthma exacerbations and poorer improvement of FEV1	Telleria et al., 2008
*ALOX5*	Tandem repeats in the *ALOX5* core promoter	USA, children and adolescents, *n =* 270	Reduced lung function and worse asthma control	Mougey et al., 2013b
*ALOX5*	Tandem repeats in the *ALOX5* core promoter	USA, adults, *n =* 252	Reduced risk of exacerbation	Lima et al., 2006
*ALOX5*	rs2115819 (A/G)	USA, adults, *n =* 577	Better response to montelukast and zileuton	Tantisira et al., 2009
*ALOX5*	rs4987105 (C/T) and rs4986832 (G/A)	USA, adults and adolescents, *n =* 174	Better response to montelukast	Tantisira et al., 2009
*LTA4H*	rs2660845 (A/G)	USA, adults, *n =* 252	Increased probability of suffering an asthma exacerbation after treatment with montelukast	Lima et al., 2006
*LTA4H*	rs2660845 (A/G)	Japan, adults, *n =* 52	Worse response to montelukast	Kotani et al., 2012
*LTA4H*	rs2540491 (C/T)	Puerto Rico and Mexico, children and adolescents, *n =* 649	Increase in FEV1 after treatment with anti-leukotrienes	Tcheurekdjian et al., 2010
*LTA4H*	rs2540487 (C/T)	Puerto Rico and Mexico, children and adolescents, *n =* 649	Increase in FEV1 after treatment with anti-leukotrienes	Tcheurekdjian et al., 2010
*LTC4S*	rs730012 (A/C)	UK, adults,*n =* 23	Improvement in lung function with zafirlukast treatment	Sampson et al., 2000
*LTC4S*	rs730012 (A/C)	Japan, adults, *n =* 349	Better response to pranlukast	Asano et al., 2002
*LTC4S*	rs730012 (A/C)	USA, adults and adolescents, *n =* 12	Better response to montelukast	Whelan et al., 2003
*LTC4S*	rs730012 (A/C)	USA, adults, *n =* 252	Decreased risk of asthma exacerbation after treatment with montelukast	Lima et al., 2006
*LTC4S*	rs272431 (G/T)	USA, adults, *n =* 577	Better lung function response to zileuton	Tantisira et al., 2009
*ABCC1*	rs119774 (C/T)	USA, adults, *n =* 252	Increase in % predicted FEV1after montelukast treatment	Lima et al., 2006
*ABCC1*	rs119774 (C/T)	USA, adults, *n =* 577	Better FEV_1_ response to montelukast.	Tantisira et al., 2009
*ABCC1*	rs215066 (G/A)	USA, adults, *n =* 577	Better Lung function response to zileuton	Tantisira et al., 2009
*SLCO2B1*	rs12422149 (G/A)	USA, adults, *n =* 489	Lower montelukast plasma concentrations	Mougey et al., 2009
*SLCO2B1*	rs12422149 (G/A)	Finland, adults, *n =* 33	No significant effect on montelukast pharmacokinetics	Tapaninen et al., 2013
*SLCO2B1*	rs12422149 (G/A)	USA, adolescents, *n =* 26	Lower montelukast plasma concentrations	Mougey et al., 2011
*SLCO2B1*	rs56837383 (C/T), rs35199625 (G/A), rs72559740 (A/T), rs12422149 (G/A), rs1621378 (C/T), rs2306168 (C/T) and rs2712807 (A/G)	Korea, adults, *n =* 24	No effect on montelukast plasma levels	Kim et al., 2013
*CYSLT1R*	rs2412222 (G/A), rs320995 (A/G), rs321029 (G/A) and rs321092 (A/G)	USA adults *n =* 252	No association with changes in FEV1 or exacerbation rates in patients receiving montelukast	Lima et al., 2006
*CYSLT1R*	rs200535935 (G/A)	Korea, children, *n =* 100	No association with clinical response to montelukast	Lee et al., 2007
*CYSLT1R*	rs773347588 (C/T)	Korea, adults, *n =* 89	Anti-leukotriene longer requirements for management of aspirin-intolerant asthma patients	Kim et al., 2007
*CYSLTR2*	rs912277 (A/G) and rs912278 (A/G)	USA, adults and adolescents, *n =* 174	Increase in PEF after treatment with montelukast	Klotsman et al., 2007
**BETA-AGONISTS**				
*ADRB2*	rs1042713 (G/A)	USA, adults, *n =* 190	Small decrease in PEF in patients who used salbutamol frequently	Israel et al., 2000
*ADRB2*	rs1042713 (G/A)	New Zealand, adults, *n =* 157	Increased number of asthma exacerbations associated with frequent use of salbutamol	Taylor et al., 2000
*ADRB2*	rs1042713 (G/A)	USA, adults, *n =* 78	Lower morning PEFR during treatment with salbutamol	Israel et al., 2004
*ADRB2*	rs1042713 (G/A)	USA, adults and adolescents, *n =* 174	No association with response to salmeterol	Bleecker et al., 2006
*ADRB2*	rs1042713 (G/A)	USA, adults and adolescents, *n =* 2,630	No association with response to salmeterol	Bleecker et al., 2007
*ADRB2*	rs1042713 (G/A)	USA, adults, *n =* 87	No association with response to salmeterol	Wechsler et al., 2009
*ADRB2*	rs1042713 (G/A)	USA, adults and adolescents, *n =* 544	No association with response to salmeterol	Bleecker et al., 2010
*ADRB2*	rs1800888 (C/T)	USA, adults, *N =* 659	Increased possibility of suffering severe asthma exacerbation in response to LABA	Ortega et al., 2014
*ADRB2*	rs1800888 (C/T)	India, adults, *n =* 398	Less response to Salbutamol in patients with persistent severe asthma	Bandaru et al., 2016
*ADCY9*	rs2230739 (T/C)	USA, children, *n =* 436	No association with salbutamol bronchodilator response	Tantisira et al., 2005
*ADCY9*	rs2230739 (T/C)	Korea, adults, *n =* 86	Improvement in FEV1 and MMEF in response to LABA associated with ICSs	Kim et al., 2011
*ARG1*	rs2781659 (A/G), rs2781663 (T/A), and rs2781665 (A/T)	USA, adults and children, *n =* 962	Lower bronchodilator response	Litonjua et al., 2008
*ARG1*	rs2781659 (A/G), rs2781663 (T/A), rs2781665 (A/T), and rs60389358 (C/T)	USA, adults, *n =* 96	Lower Bronchodilator response to beta2-agonists	Duan et al., 2011
*ARG1*	rs2749935 (T/A)	Chinese, adults, *n =* 345	No association with bronchodilator response	Sy et al., 2012
*ARG2*	rs17249437 (T/C) and rs3742879 (A/G)	Netherlands, adults, *n =* 200	Lower beta2-agonist reversibility	Vonk et al., 2010
*CRHR2*	rs255100 (A/T), rs7793837 (A/T), and rs2267715 (G/A)	USA, children, *n =* 607	Worse bronchodilator response	Poon et al., 2008
*CRHR2*	rs2284220 (A/G), rs7793837 (A/T), and rs2267716 (T/C)	USA, adults, *n =* 427	Worse bronchodilator response	Poon et al., 2008
*CRHR2*	rs73294475 (T/C)	USA, Latino children, *n =* 1782	Better bronchodilator response	Drake et al., 2014
*THRB*	rs892940 (G/A) rs892940 (G/A) and rs892940 (G/A)	USA, children, *n =* 607 USA, adult, *n =* 435 USA, adult, *n =* 155	Better bronchodilator response	Duan et al., 2013
*SPATS2L*	rs295137 (C/T)	USA, adults, *n =* 1,644	Marginally associated with a better bronchodilator response	Himes et al., 2012

The glucocorticoid receptor (GR) is situated in the cell cytoplasm. It makes up part of a heterocomplex of proteins that activate the GR (Pratt et al., 2006). The proteins comprising this heterocomplex include heat shock proteins and, among these, the heat shock organizing protein (Hop), or *STIP1* (Hawkins et al., 2009). Hawkins et al. postulated that genetic variations in the *STIP1* gene might discriminate individuals who are more responsive to therapy with corticosteroids. They found that a number of *STIP1* SNVs (Table 1) were related to the baseline FEV1 and percent predicted FEV1, and also that some SNVs correlate with the percent change in FEV1 after 4 (rs6591838 and rs223647) and 8 (rs6591838 and rs1011219) weeks of corticosteroid therapy (Hawkins et al., 2009). These findings have not been independently replicated so far.

*TBX21* is a member of the T-box gene family, which codifies for the T-bet transcription factor (Szabo et al., 2000). Association of a polymorphism in these genes and airway hyper-responsiveness (AHR) in children with asthma has been reported (Raby et al., 2006). Previously, Tantisira et al. had demonstrated that a missense *TBX21* polymorphism (H33Q) is associated with decreased airway responsiveness, as determined with PC20 (20% drop in FEV1), in children with asthma after treatment with ICSs (Tantisira et al., 2004b). Later, Ye et al. described in 52 asthmatic adult individuals that patients who were carriers of the variant form of the (H33Q) nonsynonymous *TBX21* rs2240017 polymorphism, had worse control of symptoms when treated with ICSs for 12 weeks, thus suggesting that the mentioned polymorphism is related to response to ICSs in airways of patients with asthma, and hence a potential biomarker of response (Ye et al., 2009). Subsequently, a report was published describing the association of a common SNV, designated as rs9910408, which is located about 10 kb of the *TBX21* in the 5′ area, with response to ICSs therapy. In particular, among adult asthma patients treated with ICSs, those who were carriers of the variant allele either in heterozygosity or homozygosity (that is, genotypes A/G or G/G), had a poorer improvement in bronchial hyperresponsiveness, FEV1, and quality of life, as compared to homozygous carriers of the wild-type allele (that is, A/A genotype) (Lopert et al., 2013). No conclusive explanation has been provided for such an association, and it should be borne in mind that the mentioned SNV is classified as intergenic and has no known functional consequences. However, taking together the apparent discrepancies between the findings reported by Tantisira et al. (2004a) and Ye et al. (2009), further studies are required before considering the *TBX21* gene as a strong candidate for pharmacogenomics studies.

In 2011, Tantisira et al. carried out a Genome-Wide Association Study (GWAS) in the Childhood Asthma Management Program (CAMP) population, and identified 13 SNVs strongly associated with changes in FEV1 after ICSs use. These SNVs included the SNV designated as rs37972, which corresponds to a non-coding exon variant in the **glucocorticoid-induced transcript 1 gene (*GLCCI1*)** (Tantisira et al., 2011). This variant is very common (with minor allele frequencies ranging from 18 to 51% in major human populations). An independent study carried out on a Chinese population (Izuhara et al., 2014) confirmed the association of the SNV rs37972 with a worse response to ICSs. Although the mentioned SNV has no known functional impact, the authors claim that the association could actually be due to linkage with another SNV, designated as rs37973. This latter SNV is an intronic variant located in a regulatory region and is related to a decrease in gene expression (Tantisira et al., 2011). The association of ICSs response with the SNV rs37973 was further confirmed in another study carried out in a Japanese population (Izuhara et al., 2014) and in two Chinese populations (Hu et al., 2016; Xu et al., 2017). These studies concluded that, among patients under ICSs treatment, the *GLCCI1* SNV rs37973 is related to lung functionality. Nevertheless, in a large study group of Caucasian asthmatic subjects who were treated with fluticasone furoate or fluticasone propionate, no association of the rs37973 SNV with FEV1 changes was observed (Hosking et al., 2014), and a recently published study carried out in a European population neither confirmed the initial association between *GLCCI1* SNV rs37972 and a poor response to ICSs (Rijavec et al., 2018). Therefore, the relevance of *GLCCI1* rs37973 as a predictor of ICSs response remains to be confirmed.

In 2012, Tantisira et al. carried out another GWAS study seeking additional factors for ICSs response in patients with asthma. They identified a novel pharmacogenetics locus, a transcription factor known as the ***T gene*** (Tantisira et al., 2012). In this study, three *T* SNVs (rs1134481, rs2305089, and rs3099266) were associated with FEV1 response to ICSs therapy. The SNV rs1134481 is an untranslated variant located in the 3′ area of the gene, the SNV rs2305089 is a missense variant (G177D), and the SNV rs3099266 is an untranslated variant located in the 5′ flanking region of the *T* gene. After including covariates in the statistical analyses, all the mentioned SNVs, when present as the homozygous wild-type genotype, were associated to significant differences in FEV1 as compared to patients with who were heterozygous or homozygous for the variant allele. No studies attempting to replicate these findings have been published thus far.

In 2007, multivariable models demonstrated an association between a ***FCER2***SNV (rs28364072) with severe asthma exacerbations in children treated with ICSs (Tantisira et al., 2007). Two years later the same authors published a study with the same children included in the previously mentioned CAMP population. They analyzed different variables potentially related to two different outcomes: exacerbations and poor FEV1 response, and their findings confirmed the relevance of the rs28364072 polymorphism (Rogers et al., 2009). Some years later, European investigators obtained similar results in a large group of patients including two populations of asthmatic children from the Netherlands (Koster et al., 2011). No studies contradicting these independent findings have been reported, and overall findings suggest that the rs28364072 SNV, which is located within the splice region of exon 8 of the *FCER2* gene, might be a promising biomarker of response to ICSs.

Moffatt et al. reported that several genetic variants of the **Orosomucoid like-3 (ORM1-like protein 3;** ***ORMDL3*)** gene determined asthma susceptibility in 994 asthmatic children. These included 10 SNVs in the GWAS phase and 9 in the replication phase, and many of these SNVs were associated with ORMDL3 transcript abundance (Moffatt et al., 2007). Subsequently, a study was performed in 311 children with mild or moderate persistent atopic asthma to test whether an intergenic *ORMDL3* SNV not identified in the GWAS study, designated as rs2872507, could influence the response to ICSs. This study concluded that, in carriers of the minor allele in homozygosity (AA genotype), FEV1 improved to a greater extent as compared to patients with different (A/G or GG) genotypes (Berce et al., 2013). A study with 110 asthmatic children confirmed that another SNV, designated as rs72821893, which was located in the same chromosomal area, is associated with ICSs treatment response (Leusink et al., 2016). Although both studies point to variants in the vicinity of the *ORMDL3* gene as putative markers of ICSs response, further studies are required to elucidate the putative mechanisms underlying the association of a gene variant that is intergenic (rs2872507) and another variant which is located at a considerable distance (about 830 Kb) from the *ORMDL3* gene.

It has been reported that a genetic variation in the vascular endothelial growth factor A (encoded by the ***VEGFA***gene), designated as rs2146323, is associated with ICSs response in children with asthma. This association is consistent with the fact that the vascular endothelial growth factor is elevated in asthma patients. The study in question revealed that individuals who were homozygous for the minor allele (AA genotype) have a better FEV1 improvement than patients with other (AC or CC) genotypes (Balantic et al., 2012). However, the rs2146323 SNV is located in a noncoding exon area, and therefore the functional implications of this SNV are uncertain.

Loci important for ICSs metabolism affecting pharmacokinetics might also produce variability in response to ICSs. A candidate gene study of cytochromes P450 (CYP) genes 3A4 (***CYP3A4***), 3A5 (***CYP3A5***), and 3A7 (***CYP3A7***) in children with asthma identified a potential association of improvement of symptoms controls with ICSs in carriers of the variant allele CYP3A4^*^22. This allele is characterized by the presence of a rare intronic SNV, designated as rs35599367, and has been related to decreased enzyme activity. The study reported that carriers of the variant genotype had better asthma control after ICS therapy with fluticasone (Stockmann et al., 2013).

### Anti-leukotrienes Agents

Anti-leukotriene agents are controller medications for asthma although, as monotherapy, they have less efficacy than ICSs in patients with persistent asthma (Hay et al., 1995). Therefore, anti-leukotrienes are habitually used as an adjuvant treatment for persistent asthma. The genes related to synthesis and the signaling pathways (notably, receptors) of Cysteinyl leukotrienes (CysLTs) are highly variable (Chauhan and Ducharme, 2012). Therefore, elucidation of pharmacogenetics biomarkers in leukotrienes pathways holds great promise to identify individuals more prone to have a positive response to anti-leukotriene-based therapy.

Cell membrane phospholipid-associated arachidonic acid, liberated by cytosolic phospholipase A2 after cell activation, produces CysLTs. The enzyme 5-lipoxygenase (5-LO), also called arachidonate 5-lipoxygenase ALOX5, converts arachidonic acid to 5-hydroperoxy-eicosatetraenoic acid (5-HPETE), and leukotriene A4 (LTA4) (Kanaoka and Boyce, 2004). This important enzyme, involved in the synthesis of CysLts, is encoded by the ***ALOX5***gene, located in 10q11.21 (Lima et al., 2006). In 1999, in a study of 221 asthma patients carried out by Drazen et al. an *ALOX5* microsatellite polymorphism was tested against the response to ABT-761, a potent and selective inhibitor of ALOX5. FEV1 increased by 18.8 ± 3.6% and by 23.3 ± 6% in homozygotes (repeat length = 5; *n* = 64) and heterozygotes (*n* = 40), respectively. Nevertheless, variant homozygotes (non-5 repeats; *n* = 10) showed a mild decrease in FEV1. Therefore, wild-type or heterozygous patients presented a significantly greater change in FEV1 than patients with the mutant genotype (Drazen et al., 1999).

A few years later, Fowler et al. also investigated the possibility that polymorphisms in the promoter of the *ALOX5* gene could influence leukotriene receptor antagonist response. They genotyped 52 patients, 40 of which were homozygous wild-type (allele 5/5) and 12 heterozygous (11 were 5/4 and one 5/6) but in that population, no homozygous mutant individuals were identified (Fowler et al., 2002). In agreement with Drazen et al. (1999), this study did not detect differences in bronchodilator response (BDR) [FEV1, forced mid-expiratory flow rate (FEF25-75) and peak expiratory flow rate (PEFR)] between the homozygous wild-type and heterozygote individuals. Similar results to these two previous studies were replicated in a subsequent study of 61 patients with moderate persistent asthma treated during 6 months with montelukast. Thirty-two of those patients (52.5%) were homozygous (5/5), 17 (27.9%) were heterozygous (4/5), and 12 (19.7%) were homozygous (4/4). The conclusion was that a decrease in the number of asthma exacerbations, an improvement in FEV1 and lower use of beta2 agonists was observed exclusively in those patients with (5/5) or (4/5) genotypes, but not in those with (4/4) genotypes (Telleria et al., 2008). In a recent study of a pediatric cohort with primarily African American ancestry, it was found that 103 out of 135 (76%) African American participants presented at least one variant allele in *ALOX5*. A lower control of asthma was observed in these patients, so authors concluded that the *ALOX5* genotype is related to increased leukotriene production and lower lung function (Mougey et al., 2013b). Nevertheless, the results reported in other studies are not consistent with those described above. One study in patients treated with montelukast described a 73% reduction of risk of disease exacerbation in individuals who were carriers of the variant allele (X/X and 5/X), as compared with wild-type homozygotes (Lima et al., 2006). These results suggested that mutant variants up-regulated ALOX5 activity.

Another result of the study by Lima et al. was that GG homozygotes (*n* = 6) for the *ALOX5* rs2115819 SNV presented a higher FEV1 response to montelukast (30%) compared with AA homozygotes (*n* = 11) and heterozygotes (*n* = 38) in which FEV1 only increased by 4.4% and 2.0%, respectively. The same research group obtained similar results investigating the *ALOX5* rs2115819 SNV and clinical response to the 5-lipooxygenase inhibitor zileuton. The homozygous (AA) subjects had the least improvement following therapy with both drugs, whereas heterozygous subjects had intermediate improvement levels. Hence, the authors concluded that the SNV rs2115819 was related to response to montelukast and zileuton (Tantisira et al., 2009).

In 2007, Klotsman undertook another research study on asthma patients randomized to montelukast in a clinical trial seeking associations between variability in relevant genes corresponding to the *ALOX5* biosynthetic pathway and receptor, and pulmonary function (Klotsman et al., 2007). Genotype-phenotype associations were identified for the *ALOX5* markers rs4987105 and rs4986832. Patient carriers of *ALOX5* genotypes with the variant alleles showed a better response to montelukast. Further replication studies are required to confirm such associations.

Copy number variations have been described for this gene, and are related to drug allergy, but the putative role of these variations in the response to asthma treatment has not been analyzed (Plaza-Serón M del C et al., 2016).

Leukotriene A4 Hydrolase (LTA4H) converts the unstable LTA to the pro-inflammatory chemoattractant leukotriene B4 (LTB4) (Drazen et al., 1999). The *LTA4H* gene is located on chromosome 12q22 (Haeggström, 2000). Some investigations have supported the role of *LTA4H* gene SNVs in asthma and atopy susceptibility in different adult and children populations (Holloway et al., 2008; Via et al., 2010; Almomani et al., 2013). In 2006, Lima et al. studied 252 participants with poorly controlled mild to moderate persistent asthma randomized to montelukast treatment as participants in a large clinical trial. They searched for variability in genes corresponding to the leukotriene pathway and the potential associations to clinical response to this drug. After treatment with montelukast, carriers of the G allele of rs2660845 had a 4- to 5-fold increased probability of suffering an asthma exacerbation compared with AA homozygotes, although the mechanism to explain this association is unknown (Lima et al., 2006). Results were replicated in a Japanese study of 21 asthma patients [men: 12, women: 9, age: 62.7 ± 15.2 (mean ± SD)] with montelukast treatment for 4–8 weeks (Kotani et al., 2012). In 2010 Tcheurekdjian et al. carried out a study in two Latin populations (356 Puerto Ricans and 293 Mexican asthmatic patients, with persistent asthma in 67.2% of them) with the primary endpoint of evaluating the effect of *LTA4H* gene polymorphisms on the association between leukotriene modifier use and bronchodilator responsiveness to albuterol. After albuterol administration, a clinically significant increase in percent change in FEV1 of 7.10, 10.06, and 10.03% was found in carriers of the minor *LTA4H* rs2540491 allele (either in heterozygosity or homozygosity), and in carriers in heterozygosity of the wild-type rs2540487 allele, respectively. Nevertheless, when stratified by ethnicity, Puerto Rican participants followed the same pattern in augmentation of bronchodilator responsiveness by leukotriene modifiers, but the Mexican participants did not (Tcheurekdjian et al., 2010). Overall, findings regarding *LTA4H* require further, independent, confirmation.

LTC4 synthase **(LTC4S)** adducts glutathione at the C-6 position of LTA4 converting LTA4 in leukotriene C4 (LTC4). There is evidence that the gene encoding ***LTC4S***is polymorphic (Chauhan and Ducharme, 2012). One initial study with few patients with persistent asthma demonstrated that patients carrying the *LTC4S* rs730012C allele in heterozygosity or homozygosity generated approximately triple LTC4 compared to non-carriers. Two parameters of lung function [FEV1 and forced vital capacity (FVC)] improved in patients treated with zafirlukast with the variant genotypes. The results of this study suggest that the pathophysiology of asthma is related with an increased CysLTs synthesis in the 56% of patients with the variant *LTC4S* genotype, indicating that this subgroup of patients could represent a target group of a better response to anti-leukotriene therapy (Sampson et al., 2000). Similar results were replicated in 3 subsequent studies. In the first of these, Japanese patients were evaluated with respect to response to pranlukast. It was observed that carriers of the *LTC4S* rs730012C had a better response to pranlukast in comparison with carriers of the *LTC4S* rs730012A allele in homozygosity, regarding FEV1 improvement (Asano et al., 2002). In the second study, pediatric patients were evaluated after montelukast treatment. Heterozygote *LTC4S* patients responded better to montelukast compared to A/A homozygotes, regarding changes in the fraction of exhaled nitric oxide (FENO) (Whelan et al., 2003). In the third study, in patients receiving montelukast there was an association with decreased risk of asthma exacerbation associated to the *LTC4S* rs730012C allele, as compared with the group of patients with the *LTC4S* rs730012A allele in homozygosity (Lima et al., 2006). A subsequent study reported the association of the *LTC4S* rs272431 SNV with a better lung function response to the anti-leukotriene zileuton (Tantisira et al., 2009). Copy number variations are uncommon in this gene, but the putative role of these variations in the response to asthma treatment has not been analyzed (Plaza-Serón M del C et al., 2016). Overall, a number of independent studies support a role for *LTC4S* gene variations as potential biomarkers of response to anti-leukotriene agents.

The multidrug resistance protein 1 **(MRP1**) transports LTC4 to the extracellular space. ***ABCC1* gene** on chromosome 16p13.1 encodes Human MRP1 (Cole, 2014). An increase in % predicted FEV1 in participants treated with montelukast for 6 months was associated with the genotype of the *ABCC1* intronic variant rs119774, in a study carried out by Lima et al. that included 252 participants with poorly controlled mild to moderate persistent asthma. In fact heterozygotes (CT) presented a substantial 24% increase in the predicted FEV1, as compared with the almost negligible 2% increase observed in carriers of the C allele in homozygosity (Lima et al., 2006). Tantisira et al. obtained similar results in their study of 577 patients with moderate asthma receiving zileuton (Tantisira et al., 2009). These authors also described the association of rs215066 in *ABCC1* with lung function response to zileuton. The two studies on the SNV rs119774 (C/T) are consistent and add up to more than 800 patients. Such a promising association deserves further investigation.

The organic anion transporting polypeptides 2B1 (**OATP2B1)** is encoded by the *SLCO2B1* gene. This protein is widely expressed in several organs and tissues (Hagenbuch and Meier, 2004). Mougey et al. identified two transporters responsible for montelukast absorption: OATP2B1 and OATP1A2 (Mougey et al., 2009). These authors reported an association between plasma concentrations of montelukast and the non-synonymous SNV *SLCO2B1* rs12422149 (G/A) which causes the amino acid substitution R312Q. They observed that plasma concentrations in subject homozygotes were higher in comparison with those observed in heterozygotes. Moreover, in homozygotes, the Asthma Symptom Utility Index scores improved after 1 and 6 months of montelukast treatment in comparison with baseline scores. Hence the SNV rs12422149 could influence montelukast pharmacokinetics and drug response. These results were replicated by the same authors in another study involving adolescent patients (Mougey et al., 2011). Nevertheless, two subsequent studies on healthy subjects in Koreans and White Finnish, respectively, did not support the effects of the *SLCO2B1* rs12422149 SNV in montelukast pharmacokinetics (Kim et al., 2013; Tapaninen et al., 2013).

Two genes encode Cysteinyl leukotriene 1 (*CYSLTR1*) and 2 (*CYSLTR2*) receptors (Chauhan and Ducharme, 2012). Asthma symptoms are caused by cysLTs [LTC4, leukotriene D4 (LTD4), and leukotriene E4 (LTE4)]. CysLT actions are mediated by the cysLT1 receptor, which is present in leukocytes (Busse and Kraft, 2005). *CYSLTR1* gene variations have been related to NSAID-induced urticaria (Cornejo-García et al., 2012), and it has been postulated that genetic polymorphisms could influence the response to drugs which acts at the CYSLTR1 receptor, such as the antagonists montelukast, pranlukast, and zafirlukast (Chauhan and Ducharme, 2012), although few studies have been published in this regard.

In 2006, no association was found between genotype of a number of SNVs in the *CYSLT1R* gene and changes in FEV1, or exacerbation rates, in patients receiving montelukast (Lima et al., 2006). Similar results were obtained by Lee et al. who concluded that the *CYSLTR1* rs200535935 (G/A) genotype does not permit prediction of clinical response to montelukast (Lee et al., 2007). Kim et al. studying the montelukast dose required to achieve asthma control, demonstrated that patients with the variant genotype (CT or TT) for the rs773347588 (C/T) SNV, presented higher expression levels than those subjects with the wild-type genotype in homozygosity (CC), thus suggesting that this polymorphism could be a useful biomarker to predict leukotriene receptor antagonists response in the treatment of aspirin-intolerant asthma patients (Kim et al., 2007). Nevertheless, the functional impact of this SNV is uncertain because it is an intronic variant, and their allelic frequency is extremely low.

Polymorphisms affecting the CYSLTR2 receptor have been proposed as useful for certain patients in the future, although they are not considered as a current drug target yet (Chauhan and Ducharme, 2012). In 2002 Thompson et al. described a novel variant in the human CysLT2 receptor, in the genetically isolated highly atopic population of Tristan da Cunha, an island in the South-Atlantic ocean. This variant was clearly associated with atopy, and was also over-represented in asthmatics, although in the last case without statistical significance. Besides, the association with atopy was independent of asthma. Authors showed that LTD4 and a partial agonist called BAY u9773 were less potent in activating the receptor with this variant compared to the wild-type receptor (Thompson et al., 2003). Pillai et al. replicated these results. They demonstrated a statistically significant association between this gene variation and asthma. This variant causes a change from methionine to valine, at position 201 (M201V). These authors also observed that this variant diminished the potency of LTD4. The location of this polymorphism in the transmembrane domain 5 could produce a change in ligand-receptor interactions or a decreased ability to initiate an adequate response (Pillai et al., 2004). The association of the variant M201V with atopy and asthma has been extensively analyzed in an interesting review about cysteinyl leukotrienes pathway genes, atopic asthma and drug response (Thompson et al., 2016). There is evidence of clinically relevant pharmacogenetic effects in PEF with *CYSLTR2* (gene variants rs912277 and rs912278) in asthma patients treated with montelukast (Klotsman et al., 2007). The authors of this study concluded that *CYSLTR2* polymorphisms could cause an increase in cysteinyl-leukotriene concentration in a subset of patients, indicating a distinct asthma clinical presentation with a better response to anti-leukotriene therapy. Independent studies are required to gain more ground on this putative association.

### Beta-Agonists

Beta-agonists are the most commonly prescribed drugs for asthma, and nowadays three drug classes are available: Short-acting beta-agonists (SABA) including isoproterenol, fenoterol, levalbuterol, terbutaline, and albuterol or salbutamol, long-acting beta-agonists (LABA) such as salmeterol and formoterol and the new ultra-long-acting beta agonists (vilanterol and indacaterol) (Ortega et al., 2015).

Albuterol (salbutamol) is the ADRB2-selective drug most used for rescue from acute bronchospasm and was the first of these drugs to be widely used by asthma patients (Pera and Penn, 2016).

Rare asthma-related life-threatening events have been associated with beta-agonists. These events are responsible for the so-called beta agonist controversy that still persists today. Isoproterenol was first related to asthma mortality epidemics in the 1960s. Subsequently, fenoterol was associated with this increased mortality in the 1970s (Ortega et al., 2015). Later on, LABA was related to serious events in some patients such as asthma-related hospitalization, intubation, and even death. This resulted in a boxed warning from the Federal Food and Drug Administration (FDA) (Chowdhury and Dal Pan, 2010). Association of LABA with inhaled corticosteroids constitutes the main treatment for persistent asthma (Plaza Moral and Comité Ejecutivo de GEMA, 2016; Global Initiative for Asthma (GINA), 2018). Therefore, identification of risk biomarkers could be critical in increasing the safety of these drugs (Ortega et al., 2014).

Catecholamines act on different tissues binding with the ADRB2 that are G protein-coupled receptors, which is encoded by the ***ADRB2***gene (Drysdale et al., 2000). In the year 2000, two studies examined with similar results whether polymorphisms at codon 16 (*ADRB2*-16) causing the amino acid substitution of (G16R, rs1042713) could influence the response to regular use, as compared to as-needed use, of salbutamol. In the first of these studies, Israel et al. showed that there was a small decrease of PEF in patients homozygous for arginine (16R) who used salbutamol frequently. This effect was enhanced during a 4-week run-out period. In that period all patients returned to using as-needed salbutamol. At the end of the study it was observed that 16R/R patients who had regularly used salbutamol showed a morning PEF 30.5 ± 12.1 L/min lower than 16R/R subjects who had used salbutamol only as a rescue medication (Israel et al., 2000). The second study concluded that homozygous (16R/R) patients were more prone to developing episodes of asthma exacerbations if they used salbutamol frequently (Taylor et al., 2000). Some years later, Israel et al. replicated these findings, and concluded that the rs1042713 SNV, which conditions the G16R genotype, influences long-term response to salbutamol, and that patients who are homozygous (16R/R) could benefit from avoiding salbutamol (Israel et al., 2004). Subsequently, independent studies have examined whether the same polymorphism affects the asthma control of patients treated with LABA, but most of them conclude that treatment with LABA or combinations of LABA and ICSs does not alter asthma control, nor does it increase exacerbations in adult or in adolescent patients (Bleecker et al., 2006, 2007, 2010; Wechsler et al., 2009). Nevertheless, a recent meta-analysis in children concluded that an increased risk of asthma exacerbation was associated with the treatment of a LABA as an “add-on controller” (Turner et al., 2016) and a recent systematic review has confirmed these results, thus concluding that the *ADRB2* rs1042713 variant is associated with response to LABA increasing the risk of exacerbation only in pediatric patients, but not in adults (Slob et al., 2018a). Currently, an on-going clinical trial, called PUFFIN trial, is testing the efficacy and cost-effectiveness of *ADRB2* Gly16Arg polymorphism–genotype guided treatment in asthmatic children. In this trial, a usual care study arm has been compared with a precision medicine-guide arm. No results of this trial have been published yet (Vijverberg et al., 2017).

Another polymorphism of the *ADRB2* gene designated as rs1800888 causes the amino acid substitution T164I (Green et al., 1993). Green et al. demonstrated that beta-agonists produced a decreased stimulation of adenylyl cyclase for the mutated receptor, as compared with the wild-type *ADRB2*. Hence, the authors concluded that the interaction of salmeterol with the mutated receptor is altered, thus reducing duration of action (Green et al., 2001). Some years later, it was published that in the Copenhagen General Population Study, the same *ADRB2* gene variant was associated with decreased forced expiratory volume in 1 s (FEV1) % predicted (% pred) and FEV1/FVC: Homozygous and heterozygous carriers of the variant receptor had 7% and 1% reduced FEV1 % pred and 6% and 1% reduced FEV1/FVC, respectively, as compared to patients with the wild-type receptor. Authors proposed that the general population with the variant receptor had reduced lung function and more risk of developing Chronic Obstructive Pulmonary Disease (COPD) (Thomsen et al., 2012).

In a clinical study, Ortega et al. have corroborated the *in vitro* findings described by Green et al. They reported that the T164I *ADRB2* variant was associated with increased odds of suffering severe asthma exacerbation requiring hospitalization in a non-Hispanic White American population. This suggests that alteration in response to LABA in this asthmatic population is caused by the T164I *ADRB2* variant (Ortega et al., 2014). These authors propose that genotyping for this variant be included in standard guidelines for asthma treatment, in order to choose the best therapeutic option for carriers of this variant with poor asthma control. A recent study has reported in a South-Indian population that the T164I *ADRB2* polymorphism was not considered as a risk biomarker for asthma, but that it could be a predictive biomarker for salbutamol refractoriness in patients with persistent severe asthma (Bandaru et al., 2016). In summary, several independent studies associate the T164I *ADRB2* variant with an alteration response to SABA and in some studies, with LABA, and the detection of this mutation could make it a candidate to be included in future clinical practice guidelines for asthma patients.

Activation of ADRB2 (that are G (alphas)-coupled receptors) transmits signaling with the mediation of **Adenylyl cyclase (ADCY)**. Small et al. characterized the polymorphism in the coding region of the *ADCY9* gene rs2230739 (T/C) causing the I772M amino acid substitution. This substitution results in a loss of function compared with wild-type *ADCY9*. The 772M variant of the ADCY9 enzyme has lower adenylyl cyclase activity, and also decreased activity after stimulation of G(alphas) (Small et al., 2003). In 2005, Tantisira et al. demonstrated *in vitro* that the same polymorphism was associated with a glucocorticoid-specific increase of BDR to albuterol. The same effect has been observed in children with asthma, a two-drug interaction being reported in asthma pharmacogenetic studies (Tantisira et al., 2005). Similar findings were described in a Korean study, in which authors reported that *ADCY9* gene polymorphisms could affect response to LABA associated with ICSs (formoterol with budesonide), and also that BDR to b2-agonist used in associated therapy could be improved by interaction of two polymorphisms such us *ADCY9* I772M and *ADRB2* G16R (Kim et al., 2011). Additional independent studies are required to confirm such putative associations.

Arginase converts L-arginine into L-ornithine and urea. Two isoenzymes of arginase, namely **arginase 1 (ARG1) and arginase 2 (ARG2)**, exist. These are encoded by two different genes (*ARG1* and *ARG2*). Nitric oxide synthase (NOS) also use L-arginine as a substrate to produce nitric oxide (NO) and L-citrulline. One of the biological functions of arginase present in extrahepatic organs could be the regulation of NO levels in competing with NOS for the common substrate (Maarsingh et al., 2009). In 2008, Litonjua et al. identified three SNVs (rs2781659, rs2781663, and rs2781665) in *ARG1*, all mapped within the gene promoter, which were proposed as novel BDR determinants. These SNVs were associated with lower adjusted BDR, although only the association with the rs2781659 SNV remained significant after correction for multiple comparisons (Litonjua et al., 2008). Three years later, the same authors reported that these polymorphisms were associated with BDR in three asthmatic populations. The most common haplotype (designated as haplotype 1) was correlated with higher BDR in all studies, and the two less frequent haplotypes were associated with low BDR (Duan et al., 2011). It has also been suggested that the rs2749935 polymorphism in *ARG1* might be associated with BDR, although this putative association did not remain significant after adjustment for multiple comparisons (Sy et al., 2012). In their study of *ARG1* and *ARG2* polymorphisms, Vonk et al. reported that two polymorphisms present in *ARG2* (designated as rs17249437 and rs3742879, respectively) were associated with asthma, severe airway obstruction and with augmented airway hyper-responsiveness. Moreover, *ARG1* and *ARG2* were associated with lower beta2 agonist reversibility, but not with anticholinergic reversibility (Vonk et al., 2010).

In summary, two studies by the same research group support a role for *ARG1* SNVs in bronchodilator response and another study did not find a significant association with another SNV. It is to be noted that the strongest association was observed with the rs2781659 SNV, which is located within the regulatory region and affects transcription factor binding sites, whereas the rest of the SNVs which had no significant association with bronchodilator response, are intergenic and, therefore, their functional effect is uncertain. Regarding the *ARG2* gene, one SNV is intergenic and the other one is located in the 3' region, and neither have known functional impact. Overall findings are, therefore, promising at least for *ARG1*, although additional replication studies are needed.

#### The Corticotropin-Releasing Hormone Receptor-2

**The corticotropin-releasing hormone receptor-2**, encoded by the *CRHR2* gene (located in chromosome 7p21-p15), regulates the relaxation of bronchial smooth muscle by activation of ADCY and protein kinase A (Meyer et al., 1997; Ortega et al., 2015). In three independent cohorts, five different SNVs in *CRHR2* have been described to be associated with an acute SABA BDR. Variants present in the 5′ region were associated in each of these three cohorts, although none of the single variants were significantly associated with BDR in all of the three groups of patients, so the functional effect of these variants is probably weak (Poon et al., 2008). A previously mentioned study (Sy et al., 2012) analyzed gene-gene interactions and revealed an association between BDR and the rs2749935 SNV in *ARG1* and rs2190242 SNV in the *CRHR2* gene, although these associations did not remain statistically significant when adjusted for multiple testing. The GALA (Genetics of Asthma in Latino Americans) study carried out on 1782 Latino children with asthma, has shown that a rare variant in *CRHR2* (rs73294475) is associated with BDR, but no association with other previously published variants in this gene were found (Drake et al., 2014). Further replication studies are required to confirm these putative associations.

Thyroid hormone binds to thyroid hormone receptors (THRs) to effect its action. Two different genes encode these receptors, called thyroid hormone receptor α (*THRA*) and β (*THRB*) genes, respectively. The ***THRB* gene** is expressed as two isoforms designated as THRβ1 and THRβ2 (Ortiga-Carvalho et al., 2014). Only one study has reported that a promoter SNV, designated as rs892940, is associated with response to beta 2-agonists, in a study with asthmatic children. This association has been further replicated in two adult asthma populations (Duan et al., 2013). Genetic variants in *THRB* might affect the expression of this receptor and have effects on transcription regulation that may contribute bronchoconstriction and inflammation of the airways. Nevertheless, the exact mechanism by which *THRB* modulates BDR is unknown.

In 2012, Himes et al. carried out a GWAS on BDR in asthmatics patients. Authors showed an association with BDR and variants located near the spermatogenesis-associated serine-rich 2-like **(*SPATS2L*) gene**. The SNV with the most relevant association was rs295137, an intergenic variant, which is located about 20.5 Kb from the *SPATSL2* gene. Individuals with the TT genotype had a better BDR than those with CT or CC genotypes. Authors speculated that the TT genotype is related with a decreased gene transcription, which results in an increase in ADRB2 levels (Himes et al., 2012). Nevertheless, this association had a combined *P*-value across all studied cohorts of 9.7E-07 and this *P*-value does not meet criteria of conventional GWAS significance. Further studies are required to elucidate the way in which specific SNV affecting *SPATS2L* effects might impact ADRB2 signaling and BDR, but no more articles on this potential association have been published.

Drug metabolism of beta-agonists is considered a major factor in determining clinical response. Impaired metabolism may cause drug accumulation with the associated adverse effects or it may cause therapeutic failure if prodrugs are not efficiently converted to their active forms. It has been shown that diverse Cytochrome P450 enzymes are involved in the metabolism of formoterol, and CYP3A4 and CYP3A5 are involved in the hydroxylation of salmeterol (revised in García-Martín et al., 2013). In this respect, CYP3A5 is the principal CYP3A form in the lung (Kivistö et al., 1996), and since the above-mentioned drugs suffer pre-systemic metabolism, it is plausible that patient carriers of the gain-of-function CYP3A5^*^1 allele may have lower bioavailability, or lower therapeutic efficacy, or apparent resistance, to inhaled drugs that are CYP3A substrates, but further studies to confirm this hypothesis are necessary (García-Martín et al., 2013).

### Biologic Agents

Living organism-synthesized biologic agents are directed against a specific determinant, such as a specific cytokine or receptor. This group includes different agents targeting IgE, T helper 2 (Th2)-type and Th2-promoting cytokines, against interleukin-4 (IL-4), interleukin-5 (IL-5), interleukin-9, interleukin-13 (IL-13), interleukin-31, thymic stromal lymphopoietin (TSLP), and others (Boyman et al., 2015). Omalizumab, an anti-IgE antibody, has been available for subcutaneous SC use for more than 10 years. Subsequently, mepolizumab and benralizumab were approved for SC administration in 2015 and 2017, respectively, and reslizumab for intravenous administration in 2016 in Europe and USA (Matucci et al., 2018). These therapies are expensive, so they should only be prescribed to patients who are positive for biomarkers of responsiveness, including those based on pharmacogenetic data. These biomarkers could contribute to the practice of precision medicine, especially in uncontrolled patients (Ortega et al., 2015).

Pitrakinra is a recombinant human IL-4 variant that acts by inhibiting the interleukin-4-alpha receptor (IL-4RA) complex. A previous clinical trial had shown that this drug could substantially diminish the symptoms of asthma (Wenzel et al., 2007). A subsequent clinical trial with pitrakinra showed that, although no significant differences were observed between pitrakinra therapy and placebo, efficacy was demonstrated after ***IL4RA***genotyping in a pharmacogenetic analysis. Patients homozygous for the rs8832GG common allele that were randomized to active IL-4RA therapy suffered fewer asthma exacerbations, nocturnal awakenings and limitations in different activities compared with patients with the rs8832 A minor allele (Slager et al., 2012). A previous small pharmacogenetics study had suggested that IL4RA amino acid variations might predict therapeutic treatment response to anti–IL-4/IL-13 pathway inhibitors (Slager et al., 2010).

Recently, a *post-hoc* analysis has been published of the Dose Ranging Efficacy and Safety with Mepolizumab (DREAM) study and Mepolizumab as Adjunctive Therapy in Patients with Severe Asthma (MENSA) study. In this study, putative genetic markers were tested in subjects with severe persistent asthma who received mepolizumab, with negative results (Condreay et al., 2017).

To date, no more articles seeking pharmacogenetics associations have been published that lead to the identification of subgroups of patients with better response to biologic agents used for the treatment of uncontrolled asthmatic patients.

## Discussion

In spite of the great effort dedicated in recent years to the search of pharmacogenomics biomarkers to improve asthma therapy, compelling evidence to support such use is still insufficient. The need to obtain clinical evidence supporting the use of pharmacogenomics in asthma therapy, as is the case for other therapies, has been previously emphasized (Van Schie et al., 2011; Slob et al., 2018b). Many positive findings are preliminary and require replication by independent laboratories. In addition, in some cases replication is hampered by the different gene and SNV nomenclature used in the available studies, especially those reported many years ago. To address this issue, harmonization of the pharmacogenetics nomenclature has been proposed recently (Kalman et al., 2016) and hopefully, in the coming years, the problem will be solved. It is interesting to note that among the huge number of guidelines published by the Clinical Pharmacogenetics Implementation Consortium (CPIC®) to optimize drug therapy based on pharmacogenomics tests (up to 35 to date), none are related to the drugs used in asthma therapy (Caudle et al., 2014). Some clear clusters do exist, (see, for instance, CYSLTR1, CYSLTR2; LTC4S, LTA4H, and ALOX5 involved in the response to anti-leukotrienes), but some genes, though putatively affecting response to diverse types of drugs, are related (see, for instance, ARG1, ARG2, and VEGFA). Some of these genes are listed in the gene-drug pair assignation by CPIC, although frequently associated with other drugs not used in asthma therapy. These include the following: *CRHR1*, associated with budesonide, fluticasone and triamcinolone with a very low evidence level, *CYP3A4* associated to tacrolimus with a low evidence level, *LTC4S* associated to aspirin with a very low evidence level, *ADRB2* associated with salbutamol with a low evidence level, *CRHR2* associated with salbutamol with a low evidence level, and *CYP3A5* associated with several drugs, none of which are used in asthma. Hopefully, when replication studies confirm or reject the putative associations analyzed in this review, some additional gene-drug pairs related to asthma therapy will be added to the CPIC list and, if evidence for actionable pharmacogenetics test is confirmed, clinical practice guidelines for the use of pharmacogenomics information in asthma will become available. Especially important will be those pharmacogenetics factors that allow identification of subgroups of patients with better response to the expensive biologic agents used for the treatment of uncontrolled asthmatic patients.

In sum, with the information available to date, based on replicated studies covering a significant number of patients, at least four genes show potential for pharmacogenomics implementation in asthma therapy. These genes are related to the response to inhaled corticosteroids (*FCER2*), anti-leukotriene agents (*ABCC1* and *LTC4S*) and beta-agonists (*ADRB2*). No promising associations have been described so far for biologic agents. Further studies are also required to confirm some putative associations such as those of *GLCCI1, T* gene, and *CYP3A4* with ICSs response or *ALOX5* SNVs with anti-leukotriene response. Additionally, the role of the genetic variability of pre-systemic metabolism in the lung epithelium for inhaled beta-agonists deserves further investigation.

## Author Contributions

JG-M and CC-D wrote the first draft of the manuscript. EG-M and JA wrote sections of the manuscript. All authors contributed to conception and design of the study, manuscript revision, read and approved the submitted version.

### Conflict of Interest Statement

The authors declare that the research was conducted in the absence of any commercial or financial relationships that could be construed as a potential conflict of interest.
